# Nociceptin/OrphaninFQ Receptor Modulates the Maturation of Adult-Born Neurons in the Mouse Dentate Gyrus Under Physiological Conditions and in a Chronic Stress Model

**DOI:** 10.1007/s12035-025-05062-6

**Published:** 2025-05-26

**Authors:** Cathaline Robert, Flora D’Oliveira da Silva, Fabiola Seminara, Carlotta Martinelli, Fanny Farrugia, Chiara Sturaro, Emilie Pacary, Claire Rampon, Chiara Ruzza, Lionel Moulédous

**Affiliations:** 1 Research Center On Animal Cognition (CRCA), Center of Integrative Biology (CBI), UMR-5169, University of Toulouse, CNRS, UPS, Toulouse, France; 2 Univ. Bordeaux, INSERM, Neurocentre Magendie, U1215, F-3300 Bordeaux, France; 3 Department of Neuroscience and Rehabilitation, University of Ferrara, Ferrara, Italy; 4 LTTA Laboratory for Advanced Therapies, Technopole of Ferrara, Ferrara, Italy; 5 Centre de Recherches Sur La Cognition Animale, Université Paul Sabatier, 118 Route de Narbonne, 31062 Toulouse Cedex 09, France

**Keywords:** Nociceptin/Orphanin FQ (N/OFQ), NOP receptor, Dentate gyrus, Adult neurogenesis, Chronic stress, Dendritic spine

## Abstract

Neurogenesis persists in the adult dentate gyrus (DG) of the hippocampus, playing a critical role in memory and stress adaptation. Dysregulation of this process is implicated in cognitive deficits and depressive behaviors induced by chronic stress, while classical antidepressants are known to enhance neurogenesis. The Nociceptin/Orphanin FQ (N/OFQ) system, comprising N/OFQ and its NOP receptor, modulates memory and the stress response, yet its role in adult neurogenesis remains underexplored. Here, we investigated the impact of N/OFQ signaling on neurogenesis in the mouse DG using genetic and pharmacological approaches under basal and chronic stress conditions. In constitutive NOP receptor knockout (KO) mice, adult neurogenesis was only mildly altered, with subtle changes in neuronal maturation. However, spine density in 4-week-old adult-born DG neurons increased following conditional NOP Receptor KO in the DG. The increase was specific to stubby and thin spines, while mature mushroom spine density decreased. When NOP KO was restricted to newly born neurons, no significant differences were observed in spine density suggesting that the absence of NOP receptors in mature DG neurons influences the local environment to regulate spinogenesis in adult-born neurons indirectly. Finally, chronic corticosterone exposure impaired spinogenesis in immature neurons, and this was mitigated by systemic administration of a NOP antagonist. Our findings suggest that N/OFQ signaling indirectly regulates the maturation and connectivity of adult-born neurons through modulation of local and distal inputs. This regulation may contribute to the antidepressant and pro-cognitive effects of NOP receptor antagonists.

## Introduction

Nociceptin/Orphanin FQ (N/OFQ) is an opioid-related neuropeptide involved in the regulation of numerous physiological and pathological processes [1–3], notably stress responses [4, 5] and learning and memory [6, 7]. N/OFQ exerts its effects through a single inhibitory G protein-coupled receptor known as the Nociceptin Opioid Peptide (NOP) receptor, which is widely expressed in the central nervous system [8]. NOP knock-out (KO) mice demonstrate enhanced memory performance [9] and increased resistance to developing depressive-like behavior following acute stress [10]. Additionally, NOP antagonists have been shown to alleviate anxiety and depressive symptoms in rats exposed to unpredictable chronic mild stress [11] and in patients with major depressive disorder [12]. Despite these findings, the mechanisms by which the N/OFQ-NOP system affects these functions remain poorly understood, though the hippocampus has been proposed has a potential site of action.

Both N/OFQ and its receptor are expressed in the hippocampus [13–15]. In brain slices, N/OFQ inhibits synaptic transmission and NMDA-dependent long-term potentiation (LTP) in the glutamatergic principal cells of the dentate gyrus (DG), CA3, and CA1 subregions through both pre-synaptic and post-synaptic mechanisms [6]. Local infusions of a NOP antagonist into the dorsal hippocampus produce antidepressant-like effects in despair tests in mice [16]. Additionally, we previously demonstrated that chronic exposure to the stress hormone corticosterone (CORT) increased N/OFQ expression in the hippocampus of mice and that local NOP receptor KO in the DG/CA3 region prevented episodic-like memory deficits induced by chronic CORT [17]. In this model, the rescue of memory deficits by NOP antagonist treatment was accompanied by the normalization of neuronal activity in the CA3 region of the hippocampus [17].

NOP receptor transcripts have been detected in immature neurons in the adult DG [18], suggesting that, beyond regulating the activity and plasticity of mature hippocampal neurons, the N/OFQ system may also influence memory processes and stress adaptation by modulating adult neurogenesis. Neurogenesis continues throughout adulthood in the DG of the mammalian hippocampus [19, 20], and immature neurons in the adult DG play critical roles in the acquisition and retrieval of precise episodic-like memories [21–26]. They also contribute to fine-tuning responses and adaptations to stress [27–30]. Consequently, alterations in adult neurogenesis have been linked to cognitive deficits and depressive symptoms induced by chronic stress [31–34]. Furthermore, the efficacy of classical antidepressants is partially attributed to their ability to enhance adult hippocampal neurogenesis [35–37].

Therefore, the hypothesis that NOP antagonists produce their protective effects against stress in part by modulating adult neurogenesis in the hippocampus deserves further investigation. However, the effects of N/OFQ on neuronal development in general, and on adult neurogenesis in particular, remain poorly characterized. N/OFQ appears to exert neurotrophic effects during cerebellar development [38], but inhibits axonal regeneration after spinal cord injury [39]. In cultured hippocampal neurons, N/OFQ suppresses dendritic growth, and accordingly, N/OFQ KO mice show increased primary dendrite length and spine density [40]. These studies have predominantly focused on developmentally-born neurons, and only two have explored the modulation of DG adult neurogenesis by the endogenous N/OFQ system. We previously reported that acute administration of the NOP antagonist BTRX-246040 did not influence adult neurogenesis in either naive or stressed mice [41]. However, Vitale and colleagues demonstrated that chronic administration of the NOP antagonist UFP-101 increased the number of immature neurons in the DG of rats exposed to chronic mild stress, but not in control animals [42].

In the present study, we employed a combination of genetic and pharmacological approaches to further characterize the role of the N/OFQ system in modulating adult neurogenesis under basal conditions and in a model of chronic stress.

## Methods

### Animals

Male NOP^−/−^ mice on a CD-1 background were bred and housed in a specific pathogen-free animal facility of the University of Ferrara (LARP). Male mice with a floxed NOP receptor gene fused to Yellow Fluorescent Protein on a C57BL/6 J background (referred to as NOPRlox/lox) were obtained from Prof. Michael Bruchas (University of Washington, Seattle, WA, USA; [43]). Adult male C57BL/6 J mice were purchased from Janvier Laboratories (Le Genest St. Isle, France) and allowed to acclimatize to the animal facility for at least 1 week before the experiments began. Mice were housed 3–4 per cage, under standard conditions (21–22 °C, 55% humidity, 12-h light–dark cycle, with lights on at 8.00 a.m.) with food and water provided ad libitum. They were between 2 and 3 months old and were age-matched within each experiment.

The animal protocol was approved by the local Ethics Committee and the French Ministry of Education and Research (APAFIS#27,219–2,020,091,610,141,233). All experiments complied with the European guidelines for the care of laboratory animals (European Union Directive 2010/63/EU) and French or Italian regulations.

### Injections

#### *In Vivo* BrdU Labelling

Mice received intra-peritoneal (i.p.) injections of bromodeoxyuridine (BrdU), dissolved in saline solution (Merck, MI, Italy), at a dose of 100 mg/kg in a volume of 0.1 ml/10 g, three times over a 12-h period.

#### Injection of Viral Vectors

AAV5-hSyn-eGFP was obtained from Addgene (#50,465, Cambridge, MA, USA). AAV5-hSyn-eGFP-Cre was obtained from the University of North Carolina Virus Vector Core. They had titers ranging from 2 to 5 × 10^12^ vg/ml. Rv-CAG-tdTomato and Rv-CAG-GFP/Cre (1.5 to 3 × 10^10^ TU/ml) were produced as previously described [44] in the laboratory of Dr Nora Abrous (Inserm U1215, Bordeaux) from plasmids kindly provided by Fred Gage (Salk Institute for Biological Studies, La Jolla). Rv-hSynGW-PSD95-eGFP (2 × 10^8^ pfu/ml) were produced by Dr Maria Llorens-Martin (Centro de Biología Molecular “Severo Ochoa”, Madrid) from plasmids kindly provided by Prof. Carlos Lois (California Institute of Technology). Upon receipt, all viral vectors were stored at − 80 °C until use.

Three-month-old animals were injected i.p. with 0.1 mg/kg of buprenorphine (Buprecare, Centravet, Lapalisse, France) and anesthetized with isoflurane (4%, Vetflurane, Virbac, Carros, France) before being placed on a stereotaxic apparatus, and maintained under isoflurane (2%) throughout procedure. A protecting gel (Ocrygel, TVM lab, Centravet) was applied to the eyes. An incision of approximately 5 mm was made on the skin of the head. After drilling two holes on top of both hemispheres, viral solutions were injected using a 5 µl syringe (syringe 76–3301, needle 7803–07, Hamilton company) at the following coordinates: − 2 mm rostrocaudal and ± 1.6 mm lateral. The needle was then slowly advanced through the brain to − 2.5 mm. One microliter of the viral solution was infused at a rate of 0.1 µl per minute. The incision was then closed with a staple, and the mouse was placed in a heated recovery cage.

### Treatments

#### NOP Antagonist Administration

SB-612,111 (Axon, 1413, Groningen, The Netherlands) was used as a highly selective NOP receptor antagonist with subnanomolar affinity. The antagonist was administered i.p. at a dose of 10 mg/kg, dissolved in 10% dimethylsulfoxide (DMSO) in a 0.9% NaCl solution. The dose was chosen based on previous studies from our groups [17, 45].

#### Chronic Corticosterone Administration

The corticosterone model consisted in daily subcutaneous injections of corticosterone (20 mg/kg; 0.1 ml/10 g; C2505, Sigma-Aldrich St Quentin Fallavier, France) diluted in 10% DMSO (276,855, Sigma-Aldrich) for 2 weeks. The dose was chosen based on previous studies [46].

### Immunostaining

Mice were injected i.p. with 0.1 ml of Euthasol (400 mg/ml, Dechra, Centravet) and perfused transcardially with 0.9% NaCl for 1 min followed by 4% formaldehyde for 7 min (Prolabo, Paris, France) at a rate of 20 ml/min. The brains were postfixed in formaldehyde overnight at 4 °C, then transferred in 0.1 M phosphate buffer containing 30% (w/v) sucrose and 0.1% (w/v) sodium azide at 4 °C. Coronal sections of 30 µm (for cell counting) or 50 µm (for neuronal morphology) were cut using a SM2010 R microtome (Leica Biosystems, Nanterre, France) and stored in cryoprotectant at − 20 °C.

#### BrdU and DCX, CR or NeuN Co-labelling

To monitor adult neurogenesis in the DG of the hippocampus, three markers were used: doublecortin (DCX) to label immature neurons, calretinin (CR) which is expressed more transiently in post-mitotic adult-born neurons, and NeuN as a mature neuron marker to confirm neuronal fate [47, 48]. Free floating sections were washed three times for 15 min in phosphate-buffered saline (PBS) containing 0.25% Triton-X100 (Carl Roth, PBST), then incubated for 15 min with 10% methanol and 10% H_2_O_2_ in PBST, followed by three washes of 10 min each in PBST. They were then incubated in 2 N HCl for 1 h, followed by borate buffer for 5 and then 15 min. After another washing step, the sections were blocked in 10% normal goat serum (NGS) or normal donkey serum (NDS) for 1 h and then incubated with a rat anti-BrdU (1:400; ab6326, Abcam, Amsterdam, Netherlands) and either a rabbit anti- doublecortin (DCX) antibody (1:500; 326,003, Synaptic System, Goettingen, Germany), a rabbit anti-calretinin (CR) antibody (1:1000; 7699/3H, Swant, Merck), or a mouse anti-NeuN antibody (1:1000; MAB377, Millipore, Merck) in PBST overnight at room temperature. The sections were rinsed three times for 10 min in PBST and then incubated for 2 h with a donkey anti-rat antibody (Alexa 488; 1:500; A21208, Invitrogen, Thermo Fisher, Strasbourg, France) and either a goat anti-rabbit antibody (Alexa 555; 1:250; A21428, Invitrogen) or a goat anti-mouse antibody (Alexa 555; 1:250; A21422, Invitrogen) in PBST. The sections were then washed three more times for 10 min each in PBST. Cell nuclei were stained with Hoechst (1:10,000) during the first wash. The sections were mounted in Mowiol solution and coverslipped.

#### mCherry and GFP Labelling

Free-floating sections were washed three times for 10 min in PBST and then incubated for 1 h in 5% NDS in PBST. The sections were then incubated overnight in a solution of goat anti-mCherry antibody (1:500; SICGen antibodies AB0081-200, Cantanhede, Portugal) and/or rabbit anti-GFP antibody (1:500; Torrey Pines TP401, Thermo Fischer) with 5% NDS in PBST. The sections were rinsed three times for 10 min in PBST, followed by incubation with a donkey anti-goat antibody (Alexa 555, 1:500; A-21432, Invitrogen) and/or with a donkey anti-rabbit antibody (Alexa 488, 1:500; A-21206, Invitrogen) in PBST for 2 h. The sections were rinsed three more times for 10 min each in PBST. Cell nuclei were stained with Hoechst (1:10,000) during the first wash. The sections were mounted in Mowiol and coverslipped.

### Image Acquisition and Analysis

#### Quantification of Cell Density and Co-labelling

Cell counting was performed manually in one out of every 12 sections throughout the entire DG using Mercator software (Explora Nova, La Rochelle, France) at × 40 magnification with a Leica DM6000 B fluorescence microscope (Leica, Nanterre, France). To calculate the density of DCX and BrdU-immunoreactive cells (DCX^+^ and BrdU^+^), the total surface area of the DG granular cell layer was measured using Hoechst labelling with the Mercator region tool. Double immunolabelling for DCX/CR/NeuN and BrdU was determined by colocalization of BrdU-labelled nuclei and DCX/CR-labelled somas or NeuN-labelled nuclei in the *z*-axis.

#### Density of Dendritic Spines and Dendritic Arborization Analysis

Image acquisition was performed with a confocal scanning microscope (Leica SP8 DM6000) using the LAS-X software (Leica). Images were taken in the dorsal hippocampus in resonant mode, with a resolution of 1024 × 1024, a pinhole of 0.5–1 airy unit, an automatically optimized z-step, and a 552 laser at 2%. For dendritic spine analysis, images were acquired at × 63 magnification with a zoom of 6, and a signal averaging of 4 was applied in lines and frames. Images were taken in the middle molecular layer (mML) of the upper blade of the dorsal dentate gyrus. For dendritic arborization analysis, a magnification of × 40 was used without zoom. Signal averaging of 4 was applied in lines and frames.

After acquisition, the files were submitted to deconvolution using the Good’s MLE algorithm and the “theorical” PSF mode of Huygens Professional software (Scientific Volume Imaging, Hilversum, Nederlands). The images were analyzed in 3D with Imaris XT software (Bitplane AG, Oxford instruments, Abingdon-on-Thames, UK). Dendritic segments and whole cells were analyzed using the Imaris Filament module. For spine analysis, the total filament fragment was traced and spines were analyzed with the “detect spines” option. For the subtype spines analysis, the “classify spines option” was used detecting spines as stubby if they were within 0.4 µm of the dendritic shaft, as thin if the volume of the neck or head of the spine did not exceed 0.3 µm^3^, and as mushroom for spines outside of these criteria. For dendritic arborization, each neuron was traced from the starting point (cell body) to the end of every dendritic segment. The total dendritic tree of interest was then obtained, from which the total dendritic length was extracted. Its complexity was determined through Sholl analysis, based on quantifying the number of intersections between dendrites and the circumference of spheres with a radius increment of 10 μm starting from the soma.

### Data Analysis

Sample size was chosen based on previous experiments from our team. All statistical analyses were performed with GraphPad prism 8.0 software (GraphPad Software, La Jolla, CA, USA) or R software version 4.0.2 (R Core Team, 2020) for permutation tests. Normality was assessed with the Shapiro–Wilk test, and a Fisher test was conducted to confirm the equality of variances. Statistical tests were then chosen accordingly. Comparisons of means (*k* = 2) were performed using a Student test when possible; otherwise, a Mann–Whitney test was used. For comparisons of means (*k* > 2), a one-way ANOVA was performed followed by Tukey’s post hoc test, or Kruskal–Wallis followed by Dunn’s post hoc test. For comparisons with more than two factors, a two-way ANOVA for independent data or a two-way ANOVA for repeated measures was performed when applicable, followed by Sidak’s multiple comparison test. If normality or homogeneity of variances were not respected, a non-parametric test was performed with the R software. A linear model (LM) was created to analyze the effect of each independent variable and the effect of their interaction on the measured variable. The LM was then tested with a permutation test. The *p*-value of this test was calculated by comparing the original statistical test to the distribution of the permutation test (obtained after 10,000 permutations). R software analyses were performed using the packages: car [49], lme4 [50], lmerTest [51], nlme [52], and performance [53]. Post hoc comparisons were performed using a sequential Bonferroni correction on the alpha level, applying either the Holm or Benjamini and Hochberg method.

Differences were considered statistically significant when *p*-value < 0.05. Graphs were generated with GraphPad Prism 8.0 software. Data are presented as mean ± SEM.

## Results

### Adult Hippocampal Neurogenesis in Constitutive NOP Receptor KO Mice

In order to study adult neurogenesis in the DG of constitutive NOP receptor KO mice, 3-month-old NOP^−/−^ mice and their WT littermates were injected with BrdU to label dividing cells and euthanized 1, 14, and 28 days later.

**Fig. 1:**
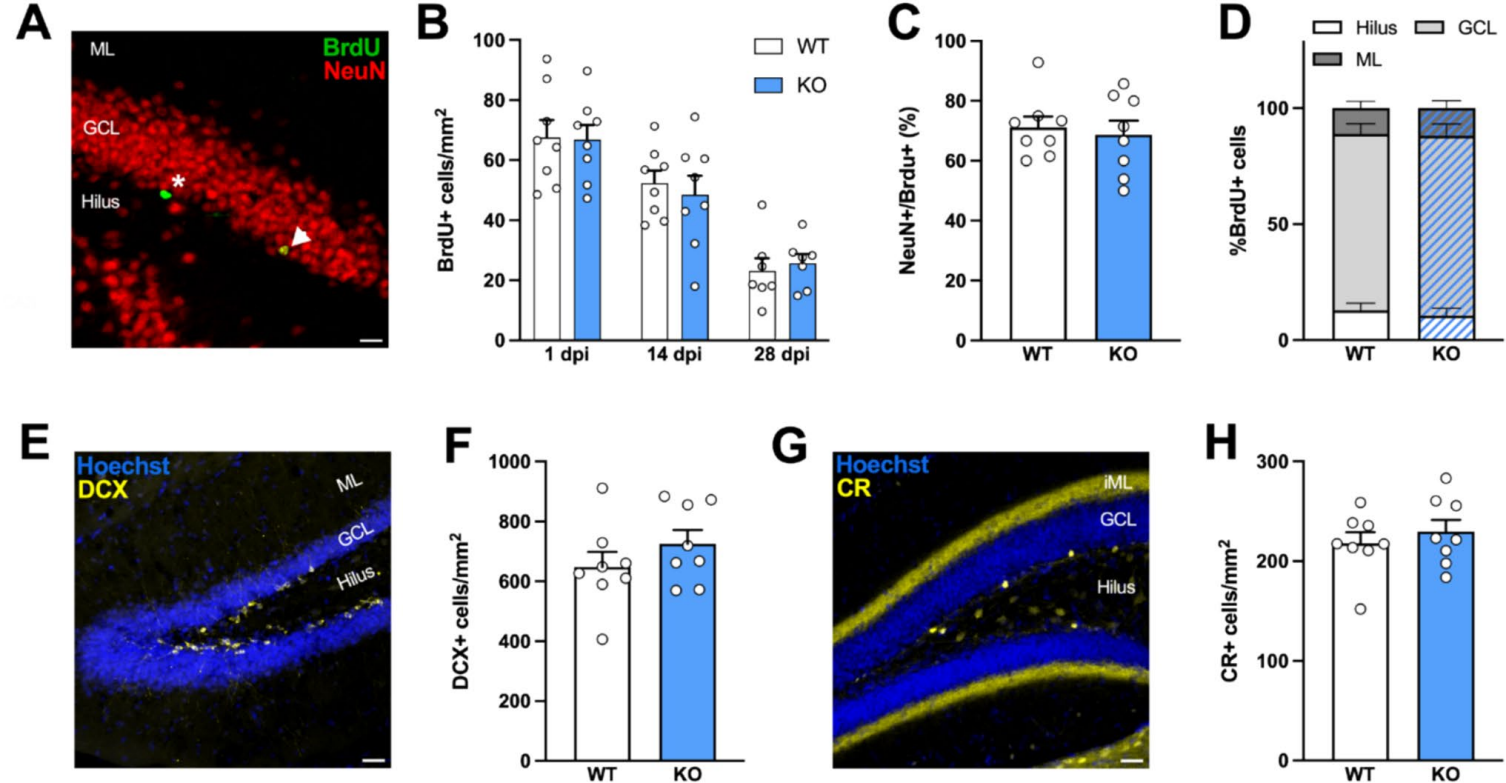
Effect of NOP receptor constitutive KO on cell proliferation, fate and survival. **A** Representative image of BrdU/NeuN double labelling. Arrowhead, BrdU+ cell expressing NeuN; Asterisk, BrdU+ cell not expressing NeuN. GCL, granular cell layer; ML, molecular layer. Scale bar = 20 µm. **B** Density of BrdU-positive cells 1, 14, and 28 days post-injection (dpi). **C** Percentage of 28-day-old BrdU-positive cells expressing NeuN. **D** Distribution of BrdU-positive cells in the layers of the DG. GCL, granule cell layer, ML, molecular layer. **E** Representative image of DCX labelling. Scale bar = 10 µm. **F** Density of DCX-positive cells in the DG. **G** Representative image of CR labelling. iML, inner molecular layer. Scale bar = 10 µm. **H** Density of CR-positive cells in the DG. *n* = 7–8 mice per group. Data are expressed as mean ± SEM

As expected, the density of BrdU-labelled cells in the DG decreased significantly over time. Moreover, it was similar between genotypes, indicating that the absence of the NOP receptor does not affect the proliferation and survival of adult-born cells (Fig. 1A, B; two-way ANOVA; Interaction, *F*_2,40_ = 0.2054, *p* = 0.8152; Time, *F*_2,40_ = 36.48, *p* < 0.001; Genotype, *F*_1,40_ = 0.02647, *p* = 0.8716). In addition, NOP receptor KO did not affect the overall rate of production of new neurons since the percentage of 4-week-old cells expressing the mature neuronal marker NeuN (Fig. 1C; Student *t*-test; *t* = 0.4043, *p* = 0.6921) and the distribution of those cells within the subregions of the DG (Fig. 1D; two-way ANOVA; Interaction, *F*_2,42_ = 0.1417, *p* = 0.8683; Layer, *F*_2,42_ = 205.1, *p* < 0.001; Genotype, *F*_1,42_ = 2.710e-014, *p* > 0.9999) were unaffected. Finally, genotype had no significant effect on the density of cells expressing the immature neuron markers DCX (Fig. 1E, F; Student *t*-test; *t* = 1.14, *p* = 0.2733) and CR (Fig. 1G, H; Student *t*-test; *t* = 0.7018, *p* = 0.4943). Overall, these data demonstrate that the production, fate, and survival of newborn neurons in the DG are not affected by the absence of NOP signaling.

**Fig. 2:**
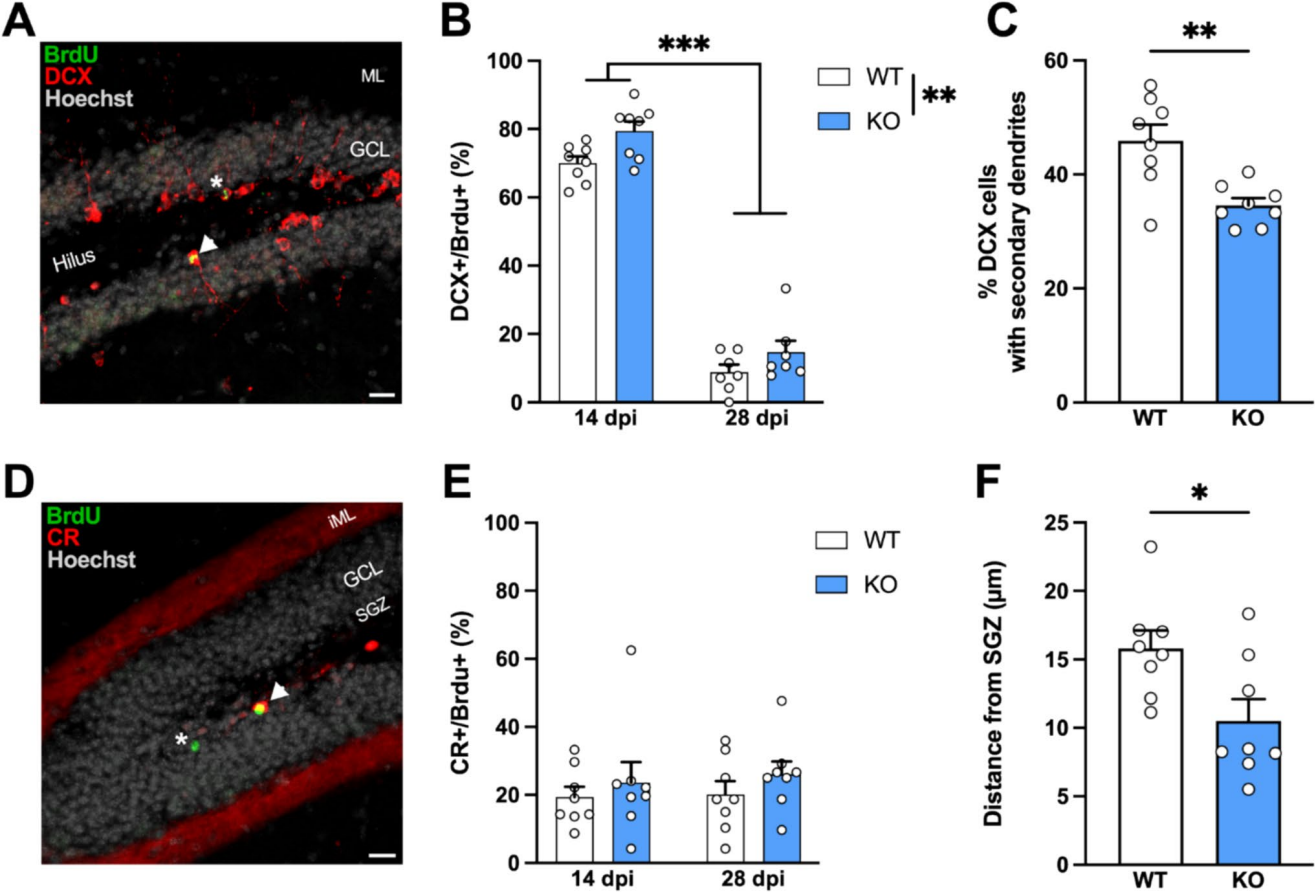
Effect of NOP receptor constitutive KO on the maturation of adult-born neurons. **A** Representative image of BrdU**/**DCX double labelling at 14 dpi. Arrowhead, BrdU+/DCX+ cell with secondary dendrites; Asterisk, BrdU+/DCX+ cell without secondary dendrites. GCL, granular cell layer; ML, molecular layer. Scale bar = 20 µm. **B** Percentage of 14- and 28-day-old BrdU+ cells expressing DCX. **C** Percentage of 14-day-old DCX+ cells with secondary dendrites. **D** Representative image of BrdU**/**CR double labelling at 14 dpi. Arrowhead, BrdU+ cell expressing CR. Asterisk, BrdU+ cell not expressing CR. iML: inner molecular layer; SGZ: subgranular zone. Scale bar = 20 µm. **E** Percentage of 14- and 28-day-old BrdU+ cells expressing CR. **F** Distance of 28-day-old BrdU+ cells from the SGZ. *p < 0.05; **p < 0.01; ***p < 0.001. *n* = 7–8 mice per group. Data are expressed as mean ± SEM

We then investigated the impact of NOP receptor KO on the maturation of the adult-born granule neurons. The percentage of newborn cells expressing the immature neuron marker DCX decreased between 14 and 28 days post-injection. Importantly, this percentage was significantly higher in KO mice than in their WT littermates (Fig. 2A, B; two-way ANOVA; Interaction, *F*_1,26_ = 0.4697, *p* = 0.4992; Time, *F*_1,26_ = 589.1, *p* < 0.0001; Genotype, *F*_1,26_ = 8.647, *p* = 0.0068). Neuronal maturation was also assessed by analyzing the percentage of immature 14-day-old DCX+ cells bearing secondary dendrites (cell indicated with arrowhead in Fig. 2A). This parameter was significantly reduced in KO mice (Fig. 2C; Mann–Whitney; *U* = 7; *p* = 0.0065). We then assessed CR, a second immature neuron marker that is expressed for a shorter period of time during the maturation process. The percentage of BrdU+ cells expressing CR did not vary significantly between 14 and 28 days post-injection, nor between WT and KO mice (Fig. 2E; Monte-Carlo permutation test; Interaction, *p* = 0.525; Time, *p* = 0.410; Genotype, *p* = 0.225). During their maturation, newborn neurons migrate from the subgranular zone (SGZ) into the granular cellular layer. The distance from the SGZ at which 28-day-old BrdU+ cells were found was significantly shorter in KO mice compared to WT mice (Fig. 2F; Student *t*-test; *t* = 2.584, *p* = 0.0216). These data suggest that the maturation process is slowed down or qualitatively altered in NOP KO mice.

### Adult Hippocampal Neurogenesis in Conditional Hippocampal NOP Receptor KO Mice

**Fig. 3:**
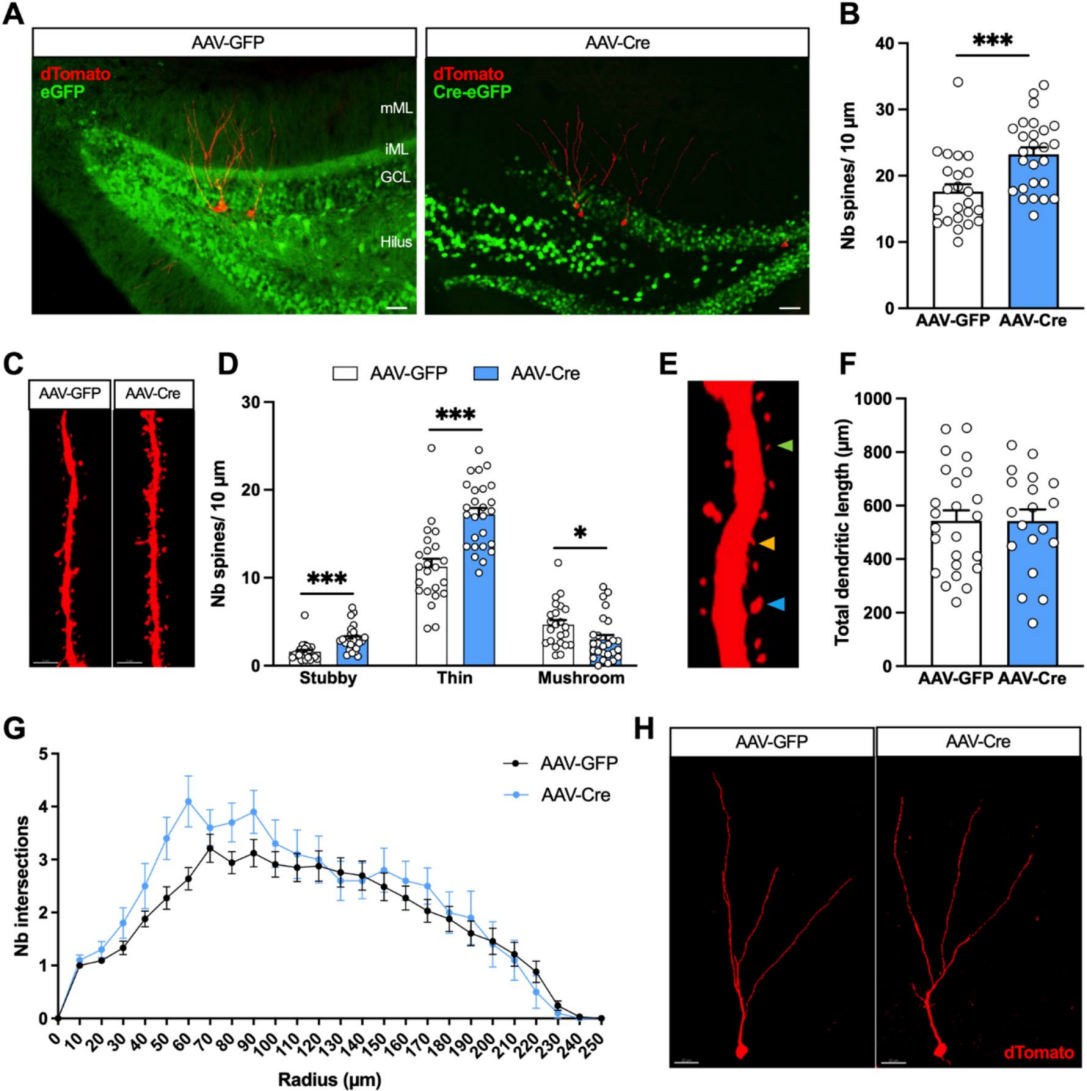
Effect of NOP receptor KO in the DG on the morphology of adult-born neurons. **A** Representative images of the DG of mice injected with Rv-CAG-tdTomato and AAV-eGFP or AAV-Cre-eGFP. GCL, granular cell layer; iML, inner molecular layer; mML, middle molecular layer. Scale bar = 50 µm. **B** Spine density of 4-week-old neurons in the mML. **C** Representative images of dendritic fragments of 4-week-old neurons. Scale bar = 3 µm. **D** Spine density of 4-week-old neurons according to the type of spines. *p < 0.05, ***p < 0.001. *n* = 23–27 dendritic fragments from 5 mice per group. **E** Image showing the different types of spines analyzed: the green arrow shows a thin spine, the orange arrow shows a stubby spine, and the blue arrow shows a mushroom spine. **F** Total dendritic length of 4-week-old neurons. *n* = 16–24 dendritic trees from 5 mice per group. **G** Sholl analysis of dendritic arborization complexity of 4-week-old neurons. **H** Representative images of the morphology of 4-week-old neurons after DG injection of AAV-eGFP or AAV-Cre-eGFP. Scale bar = 20 µm. Data are expressed as mean ± SEM

We next aimed to better characterize the contribution of the local N/OFQ system to the maturation of hippocampal adult-born neurons. To reduce NOP receptor expression, NOPRlox/lox mice were injected in the DG with AAV-hSyn-Cre-eGFP in order to induce the expression of the Cre recombinase in neuronal cells. Mice injected with AAV-eGFP were used as control. The animals also received, at the same time, injections of Rv-CAG-tdTomato vectors in the dorsal DG to transduce dividing cells, thereby labelling newly born neurons (Fig. 3A). We observed that the spine density of 4-week-old newborn neurons in the molecular layer of the DG was significantly higher in Cre expressing mice compared to the control eGFP animals (Fig. 3B, C; Unpaired *t*-test; *t* = 3.616; *p* = 0.0007). This increase was restricted to stubby and thin spines, whereas the density of mushroom spines was significantly decreased (Fig. 3D, E; Monte-Carlo permutation test; Interaction, *p* < 0.001; Spine type, *p* < 0.001; Virus, *p* < 0.001; followed by post-hoc multiple comparison test: for stubby spines, *p*(AAV-GFP vs AAV-Cre) < 0.001; for thin spines, *p* (AAV-GFP vs AAV-Cre) < 0.001; for mushroom spines, *p* (AAV-GFP vs AAV-Cre) = 0.024). Reduction of NOP receptor expression in the DG had no effect on the morphology of the dendritic tree of newborn neurons, in terms of total dendritic length (Fig. 3F; Unpaired *t*-test; *t* = 0.01409; *p* = 0.9888) and arborization complexity, assessed by Sholl analysis (Fig. 3G, H; two-way ANOVA for repeated measures; Interaction, *F*_25,1025_ = 1.440, *p* = 0.0752; Radius, *F*_4.346,178.2_ = 43.56, *p* < 0.0001; Virus, *F*_1,41_ = 1.235, *p* = 0.2729). These results suggest that the absence of the NOP receptor in DG neurons affects the spinogenesis of new neurons without significantly impacting the development of their dendritic arborization.

A close examination of Cre expression revealed that the recombinase was expressed in a large majority of DG granule cells, but in none of the dTomato labelled new neurons. This is likely due to the use of the mature neuron hSyn promoter in our AAV strategy. The observed effects are therefore non-cell-autonomous, resulting from the KO of NOP receptors in mature cells surrounding newborn neurons.

**Fig. 4:**
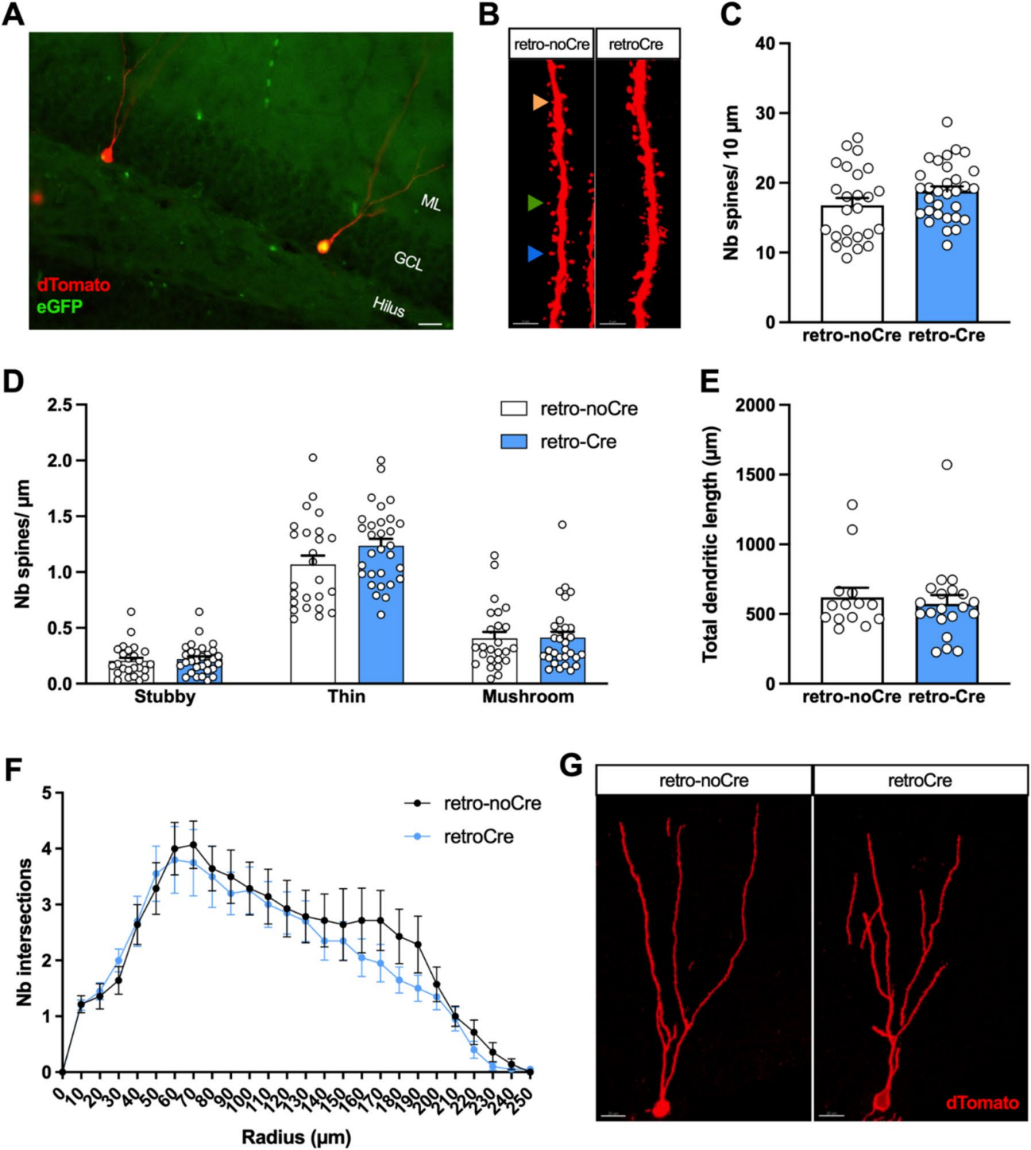
Effect of NOP receptor KO in newborn neurons on their morphology. **A** Representative image of 4-week-old neurons expressing dTomato. The neuron on the left does not co-express Cre, whereas the neuron on the right shows GPF/Cre labeling in its nucleus. GCL, granular cells layer; ML, molecular layer. Scale bar = 20 µm. **B** Representative images of dendritic fragments of 4-week-old neurons. Scale bar = 3 µm. **C** Spine density of 4-week-old neurons in the mML. The orange arrow shows a stubby spine, the green arrow shows a thin spine, and the blue arrow shows a mushroom spine. n = 24–26 dendritic fragments from 5–6 mice per group. **D** Spine density of 4-week-old neurons according to the type of spines. **E** Total dendritic length of 4-week-old neurons. *n* = 14–21 dendritic trees from 5–6 mice per group. **F** Sholl analysis of dendritic arborization complexity of 4-week-old neurons. **G** Representative images of the morphology of 4-week-old neurons expressing or not expressing Cre-eGFP. Scale bar = 20 µm. Data are expressed as mean ± SEM

To specifically address the consequence of an early KO of NOP receptor in newborn neurons, another batch of NOPRlox/lox mice were injected in the DG with a Rv-CAG-GFP/Cre vector instead of an AAV together with the Rv-CAG-tdTomato. This strategy enabled us to compare the morphology of dTomato-labelled new neurons that did or did not express the Cre recombinase in the DG of the same animals (Fig. 4A). In contrast to our previous AAV experiments, the spine density of 4-week-old neurons was similar between Cre-expressing and non-Cre-expressing cells (Fig. 4B, C; Unpaired *t*-test; *t* = 1.610; *p* = 0.1131), whatever the spine type (Fig. 4D; Monte-Carlo permutation test; Interaction, *p* = 0.241; Spine type: *p* < 0.001; Virus, *p* = 0.149). Similarly, no significant effect of conditional NOP KO was found on the morphology of the dendritic tree of new neurons, including total dendritic length (Fig. 4E; Mann–Whitney; *U* = 140; *p* > 0.9999) and arborization (Fig. 4F, G; two-way ANOVA for repeated measures; Interaction, *F*_25,800_ = 0.5319, *p* = 0.9715; Radius, *F*_3.292,105.3_ = 36.60, *p* < 0.001; Virus, *F*_1,32_ = 0.3229, *p* = 0.5739). These results suggest that NOP receptors expressed during the maturation process of new neurons do not significantly regulate neuronal development under basal conditions.

### Adult Hippocampal Neurogenesis in Mice with Systemic NOP Receptor Blockade After Exposure to CORT

**Fig. 5:**
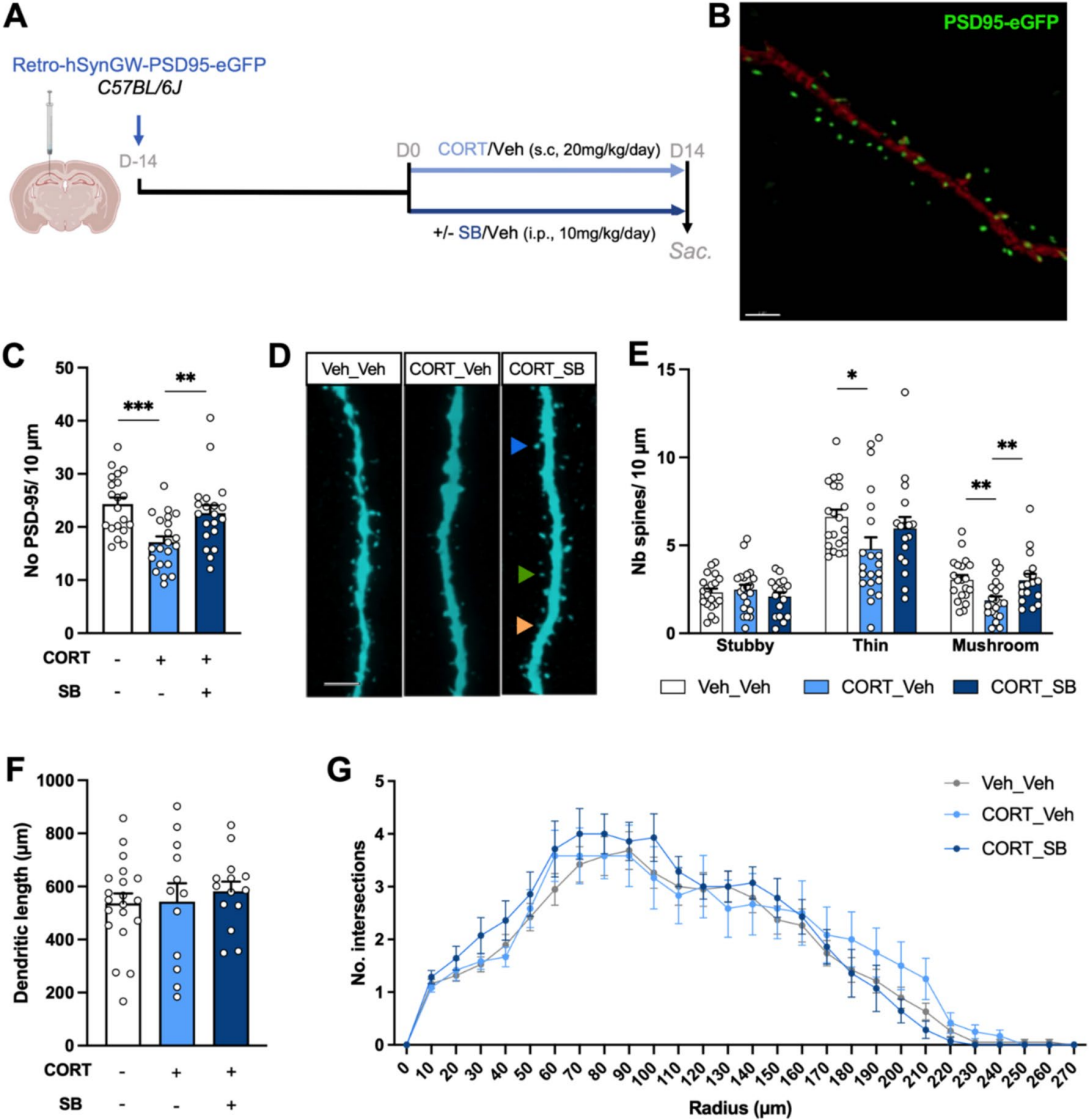
Effect of NOP antagonist treatment on neurogenesis alterations induced by repeated CORT administration. **A** Experimental design. Experimental groups included mice injected with an Rv-PSD95-eGFP vector then treated 2 weeks later with vehicle or corticosterone (CORT, s.c., 20 mg/kg/day) ± daily i.p. injection of the NOP antagonist SB-612,111. **B** Representative image of PSD95-eGFP labelling. Green, eGFP fluorescence; red, secondary antibody fluorescence. Scale bar = 2 µm. **C** Spine density of 4-week-old neurons in the mML. *n* = 20–21 dendritic fragments from 4 mice per group. **D** Representative images of dendritic fragments of 4-week-old neurons. The orange arrow shows a stubby spine, the green arrow shows a thin spine, and the blue arrow shows a mushroom spine. Scale bar = 3 µm. **E** Spine subtype density of 4-week-old neurons. *n* = 20–21 dendritic fragments from 4 mice per group. *p < 0.05. **p < 0.01. ***p < 0.001. **F** Total dendritic length of 4-week-old neurons. **G** Sholl analysis of the dendritic arborization complexity of 4-week-old neurons. *n* = 12–20 dendritic trees from 4 mice per group. Data are expressed as mean ± SEM

The data obtained so far suggest that blocking the NOP receptor may facilitate the spinogenesis of adult-born DG neurons by an indirect mechanism. To explore this further, we tested the ability of a NOP antagonist to regulate the density of dendritic spines in a pathological context. Repeated CORT administration was used to mimic prolonged stress. C57BL/6 J mice were injected with an Rv-hSynGW-PSD95-eGFP vector to label dividing cells and then exposed to pharmacological treatments 2 weeks later. These treatments included a daily subcutaneous injection of corticosterone (20 mg/kg/day) or vehicle for 14 days, coupled with an i.p. injection of either vehicle or the NOP receptor antagonist SB-612,111 (10 mg/kg/day) (Fig. 5A). We observed that repeated CORT treatment induced a decrease in the density of spines labeled with PSD95-eGFP, which was prevented in mice receiving the NOP antagonist (Fig. 5B–D; one-way ANOVA; *F*_2,58_ = 8.834, *p* = 0.0004; followed by Tukey’s multiple comparison test, *p*(CORT-/SB- vs CORT+/SB-) = 0.0005, *p*(CORT-/SB- vs CORT+/SB+) = 0.6167, *p*(CORT+/SB- vs CORT+/SB+) = 0.0093). This result indicates that blocking the N/OFQ-NOP system prevents the deleterious impact of chronic CORT on spinogenesis. To better characterize this effect, the density of the different types of spines was measured using the PSD95 signal amplified by anti-GFP immunofluorescence. Among the stubby, mushroom, and thin spines, CORT treatment significantly decreased the density of mushroom and thin spines. Co-treatment with SB-612,111 restored spine density in both types, but the effect was significant only for the mushroom subtype (Fig. 5E; Monte-Carlo permutation test; Interaction, *p* = 0.11; Virus, *p* < 0.001; Treatment, *p* = 0.01; followed by post hoc multiple comparison test, for thin spines, *p*(CORT-/SB- vs CORT+/SB-) = 0.026; for mushroom spines, *p*(CORT-/SB- vs CORT+/SB-) = 0.003, *p*(CORT+/SB- vs CORT+/SB+) = 0.006). Moreover, neither CORT treatment nor the NOP antagonist had a significant effect on the morphology of the dendritic tree of newborn neurons, including total dendritic length (Fig. 5F; one-way ANOVA; *F*_2;43_ = 0.2778, *p* = 0.7588) and dendritic arborization (Fig. 5G; two-way ANOVA for repeated measures; Interaction, *F*_54,1134_ = 0.7837, *p* = 0.8711; Radius, *F*_4.035,169.5_ = 73.44, *p* < 0.001; Virus, *F*_2,42_ = 0.3097, *p* = 0.7353).

## Discussion

In constitutive NOP receptor knockout mice, we found that the absence of NOP receptors does not drastically alter adult neurogenesis in the DG. Specifically, we observed no significant changes in cell proliferation (BrdU at 24 h), survival (BrdU at 28 days), or cell fate (DCX, CR, NeuN markers). However, neuronal maturation appeared to be subtly altered. The increase in the percentage of new neurons expressing DCX, the reduced fraction of 14-day-old neurons bearing secondary dendrites, and the decreased migration distance could result from a slower maturation process. Nevertheless, these deficits remain modest. For instance, the global density of immature DCX + cells tends to increase but this effect does not reach statistical significance. Moreover, we did not observe a significantly higher percentage of 28-day-old neurons still expressing CR, nor did we find a reduced percentage of 28-day-old cells expressing the mature neuron marker NeuN. An alternative explanation for the higher proportion of BrdU + cells expressing DCX in NOP KO mice could be an overall upregulation in DCX expression in maturing neurons. Indeed, the observed alterations in dendritic tree maturation and neuronal migration within the granule cell layer align with known functions of the microtubule-associated protein DCX [54, 55]. In any case, from a behavioral perspective, the previously reported improvements in memory performance and stress resilience in constitutive NOP KO mice [9, 10] are unlikely to result from enhanced neurogenesis, as no clear increase in adult neurogenesis was observed. However, this conclusion is limited by the use of cellular markers, which do not permit detailed analysis of neuronal maturation and connectivity. To address this limitation, we employed a more targeted approach using fluorescent proteins to specifically examine the morphology of immature neurons.

Previous studies have validated the efficacy of NOP receptor KO, notably in DG and CA3 neurons [17, 43]. We observed that reducing NOP receptor expression in a large proportion of DG neurons did not affect the dendritic arborization of 4-week-old neurons. This suggests that the reduced maturation observed in 14-day-old DCX + cells in constitutive KO mice may either be transient or indirectly mediated by NOP receptors outside the DG. Notably, NOP receptor KO in the DG resulted in increased spine density in 4-week-old neurons. Interestingly, this increase might appear inconsistent with our previous findings of impaired hippocampal-dependent memory performance in NOP receptor KO mice in this region under naive conditions [17]. However, memory improvements are not solely influenced by increased connectivity but also depend on the precise tuning of adult-born neuron excitability. Furthermore, this observed increase in spine density was restricted to stubby and thin spines, whereas mature mushroom spine density was reduced. This finding suggests that NOP receptor KO under naive unstressed conditions promotes early stages of spinogenesis but impairs the stabilization of synaptic connections, which may contribute to the observed memory deficits.

The increased spine density observed is consistent with findings in embryonically born granule cells in N/OFQ KO mice [40]. This effect may result from to the blocking of an inhibitory influence of N/OFQ on inputs that promote spinogenesis in immature neurons. Alternatively, it could stem from the loss of a direct inhibitory effect of the peptide on maturing neurons, potentially involving decreased neuronal excitability or direct regulation of their cytoskeleton (e.g., via RhoA) [40, 56].

To investigate this further, we performed targeted NOP KO in newborn neurons. Interestingly, spinogenesis was not significantly altered when Cre expression was restricted to these newly generated neurons, suggesting that the observed effects on synaptic spine development arise from the loss of NOP receptors in surrounding mature neurons [57–60]. Given that GABA remains excitatory in newborn neurons up to three weeks post-mitosis [61], and NOP mRNA is expressed not only in DG granule cells but also in some interneurons [17, 62, 63], it is plausible that N/OFQ modulates spinogenesis by inhibiting excitatory GABAergic inputs. A more detailed characterization of NOP receptor distribution across interneuron subtypes could provide valuable insights into this mechanism. Additionally, glutamate signaling is crucial for adult-born neuron development and integration [59]. Since Cre expression was confined to hippocampal cells, it is unlikely that excitatory inputs for the entorhinal cortex (EC) were affected. However, NOP KO may have reduced inhibition of local excitatory cells, such as mature granule cells or hilar mossy cells, which directly interact with immature neurons [64].

Chronic stress impairs adult hippocampal neurogenesis [34] and the therapeutic efficacy of antidepressants in rodent models of depression partially depends on adult-born neurons [37]. However, among the various stages of the neurogenesis process, spinogenesis and the functional integration of new neurons remain relatively understudied in chronic stress models [65]. Repeated administration of CORT, used to model chronic stress, disrupts neurogenesis in the DG. Chronic CORT treatment has been reported to alter cell proliferation without affecting the survival of newborn neurons or the dendritic morphology of DCX + cells [35]. However, another study reported more severe CORT effects, including reduced proliferation, survival, and DCX + neuron density after 28 days, with decreased dendritic complexity (though spine density was not assessed) [66]. Finally, chronic CORT was found to reduce activation of immature neurons during spatial memory tasks, providing indirect evidence of impaired integration into the DG network [67]. Our short CORT exposure procedure starting 2 weeks post-mitosis resulted in a mild phenotype that did not involve dendritic atrophy. However, to our knowledge, this is the first study to demonstrate the detrimental effect of chronic high CORT levels on dendritic spine density (spinogenesis) in immature neurons of the DG.

Given the interplay between CORT and N/OFQ in the modulation of hippocampus-dependent memory [17], and antidepressant properties of NOP antagonists, we next investigated whether blocking N/OFQ action could mitigate the detrimental effects of CORT on the maturation of adult-born neurons. Notably, treatment with a NOP antagonist effectively counteracted the CORT-induced reduction in spine density.

This protective effect may operate within the hippocampus, similar to the local NOP receptor KO described earlier. However, since the NOP antagonist was administered systemically in CORT-treated mice, it may have also influenced neuronal maturation indirectly by modulating extra-hippocampal inputs. The involvement of these afferents could account for the observed differences between systemic NOP blockade, which enhanced synaptic maturation (increased mushroom spine density), and local DG NOP KO, which did not produce this effect. Glutamatergic projections from the EC, cholinergic inputs from the medial septum (MS) or serotonergic projections from the dorsal raphe nucleus (DRN) are good candidates to mediate this indirect effect of the antagonist [59]. Indeed, NOP receptor activation has been shown to hyperpolarize and therefore inhibit entorhinal neurons [68]. Additionally, NOP receptors are expressed on cholinergic neurons in the MS and they reduce acetylcholine release in the hippocampus [69]. Furthermore, N/OFQ inhibits the activity of the DRN, with this inhibitory effect being amplified after swim stress [70]. Taken together, these findings suggest that an endogenous N/OFQ tone, potentially heightened under chronic stress conditions, may restrict the release of pro-maturation factors from various sources. This restriction likely contributes to deficits in production and maturation of dendritic spines in adult-born neurons.

Previous studies have demonstrated that restoring the maturation and integration of adult-born neurons can improve memory performance during normal aging [71] and in a mouse model of Alzheimer’s disease [72]. Similarly, in our chronic stress model, preserving the connectivity of immature neuron may represent one mechanism by which NOP receptor KO in the DG-CA3 region prevents CORT-induced spatial memory deficits [17]. This promotion of structural synaptic plasticity may also contribute to the antidepressant effects of NOP antagonists. A well-established connection exists between the efficacy of antidepressants and their ability to prevent deficits in dendritic spine density in mature neurons induced by chronic stress in various brain regions such as the prefrontal cortex and the hippocampus [73]. However, the case of the integration of immature neurons in the DG remains underexplored. Previous studies have shown that fluoxetine, another antidepressant drug, promotes neuronal maturation, increasing dendritic complexity in DCX + cells [74], and enhances the size of perforant path-granule cell synapses in the middle molecular layer of the dorsal DG [75]. Furthermore, repeated administration of NOP antagonist UFP-101 has been shown to produce antidepressant-like effects and increase the number of DCX + neurons in the DG under conditions of chronic stress [42]. However, this study did not examine the morphology and connectivity of these immature neurons. Further research is required to clarify how NOP antagonists influence the maturation and connectivity of adult-born neurons and their contribution to the antidepressant potential of this class of drugs. Future studies should also include animals of both sexes, as NOP receptor signaling appears to exert distinct effects in males and females exposed to stress [76]. Additionally, greater attention should be given to the spinogenesis of immature neurons in studies investigating the mechanisms of novel antidepressants in rodent models.

NOP-targeted modulators are emerging as promising therapeutics for the treatment of stress-related neuropsychiatric disorders [4, 5, 77]. In the context of traumatic stress, a single-nucleotide polymorphism within the NOP receptor gene has been linked to the severity of PTSD symptoms in a psychologically traumatized human cohort [78]. Moreover, rodent studies suggest that NOP receptor agonists may mitigate the consolidation [78] or reconsolidation [79] of traumatic memories. In contrast, for neuropsychiatric conditions with a chronic stress component, such as depression, favoring plasticity phenomena that promote resilience with NOP receptor antagonists appears to be a more effective approach. For instance, the NOP antagonist LY2940094 exhibits antidepressant-like effects not only in rodent models but also in patients with major depressive disorder [12]. Despite these promising results, the precise mechanisms by which NOP antagonists foster stress resilience remain incompletely understood. We propose that one potential mechanism involves the protection of adult-born neuron integration in the hippocampal network. Since this process is critical for both memory and mood regulation, such treatments might offer the dual benefit of preventing the cognitive and emotional deficits commonly associated with depression.

In summary, our findings demonstrate that NOP receptor signaling exerts a subtle yet significant influence on adult neurogenesis in the DG. N/OFQ indirectly regulates the spinogenesis of immature new neurons by modulating both local and distal inputs. This fine-tuning of dendritic spine formation and maturation by N/OFQ may impact the network integration of immature neurons, thereby contributing to the pro-cognitive and antidepressant effects of NOP receptor antagonists.

## Data Availability

Raw data are available from the corresponding author upon request.
